# P-135. Epidemiology and Outcomes of Infective Endocarditis in a Brooklyn Community Hospital: A Comparison of Pre- and Post-COVID Pandemic Eras

**DOI:** 10.1093/ofid/ofaf695.362

**Published:** 2026-01-11

**Authors:** May Thet Hmu Tun, Khin Htet Htet Soe, Khant Kaung Htet Lwin, Krishna Vamsy Polepalli, May Yee Lin, Sumitra Paudel, Desen Zeng, Andrew Li, Cameron Klepper, Prarath Roshni, Saira P Iqbal, Jessica Chung, Yu Shia Lin, Edward Chapnick

**Affiliations:** Maimonides Medical Center, Brooklyn, NY; Maimonides Medical Center, Brooklyn, NY; Maimonides Medical Center, Brooklyn, NY; Maimonides Medical Center, Brooklyn, NY; Maimonides Medical Center, Brooklyn, NY; Maimonides Medical Center, Brooklyn, NY; SUNY Downstate Health Sciences University, Brooklyn, New York; SUNY Downstate Health Sciences University, Brooklyn, New York; SUNY Downstate Health Sciences University, Brooklyn, New York; Maimonides Medical Center, Brooklyn, NY; Maimonides Health, Brooklyn, NY; Maimonides Health, Brooklyn, NY; Maimonides Medical Center, Brooklyn, NY; Maimonides Medical Center, Brooklyn, NY

## Abstract

**Background:**

Infective endocarditis (IE) is a severe and often life-threatening infection involving the heart valves, endocardium, or cardiac devices. Although uncommon, its incidence and mortality in the United States increased from 1990 to 2019. However, data on IE incidence and outcomes in the post-pandemic era remain limited. The use of rapid PCR diagnostics has become widespread in recent years. We hypothesize that the incidence of IE cases, hospital length of stay (LOS), and in-hospital mortality rate will increase in the post-pandemic era due to the enhanced ability of rapid PCR testing to identify pathogens, enabling earlier initiation of targeted antimicrobial therapy.

**Figure:**
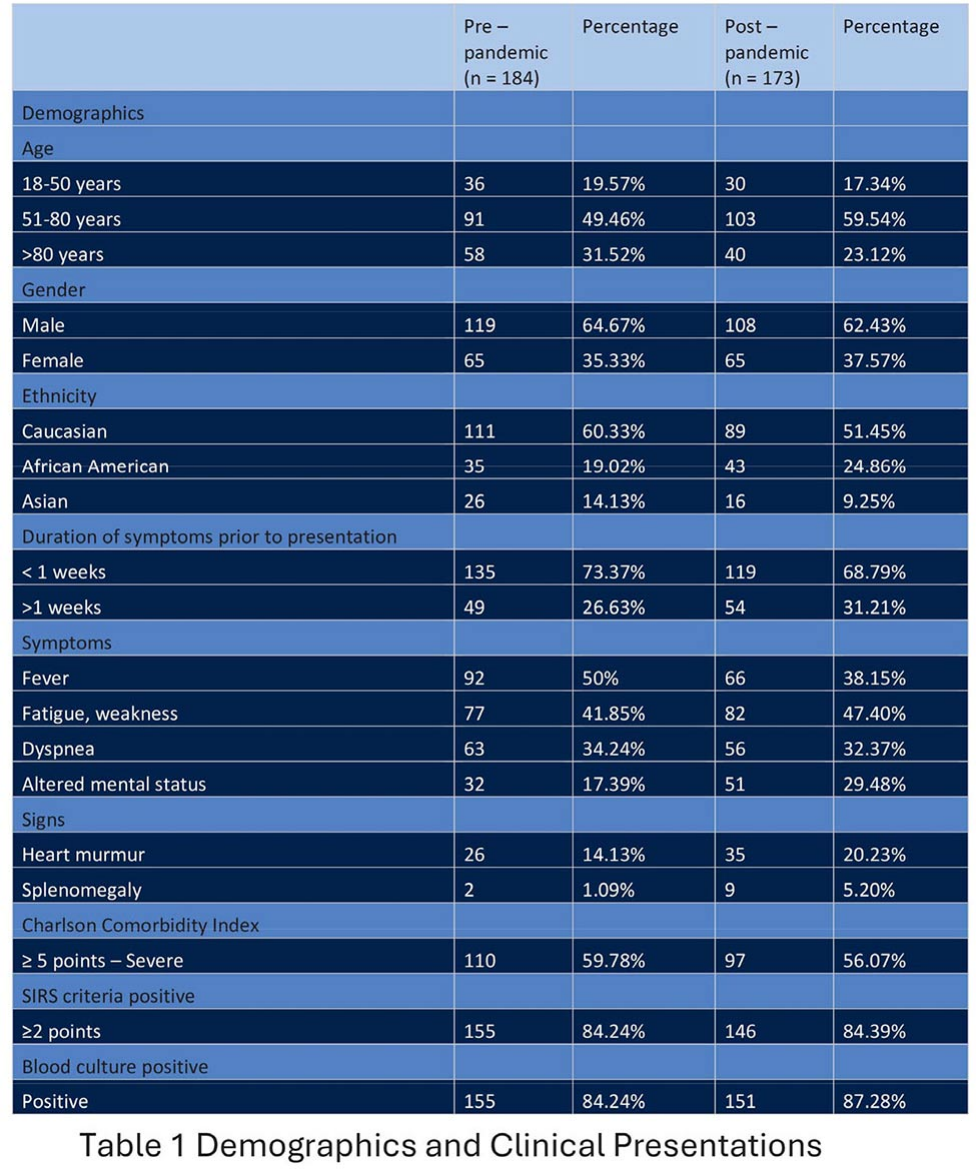

**Figure:**
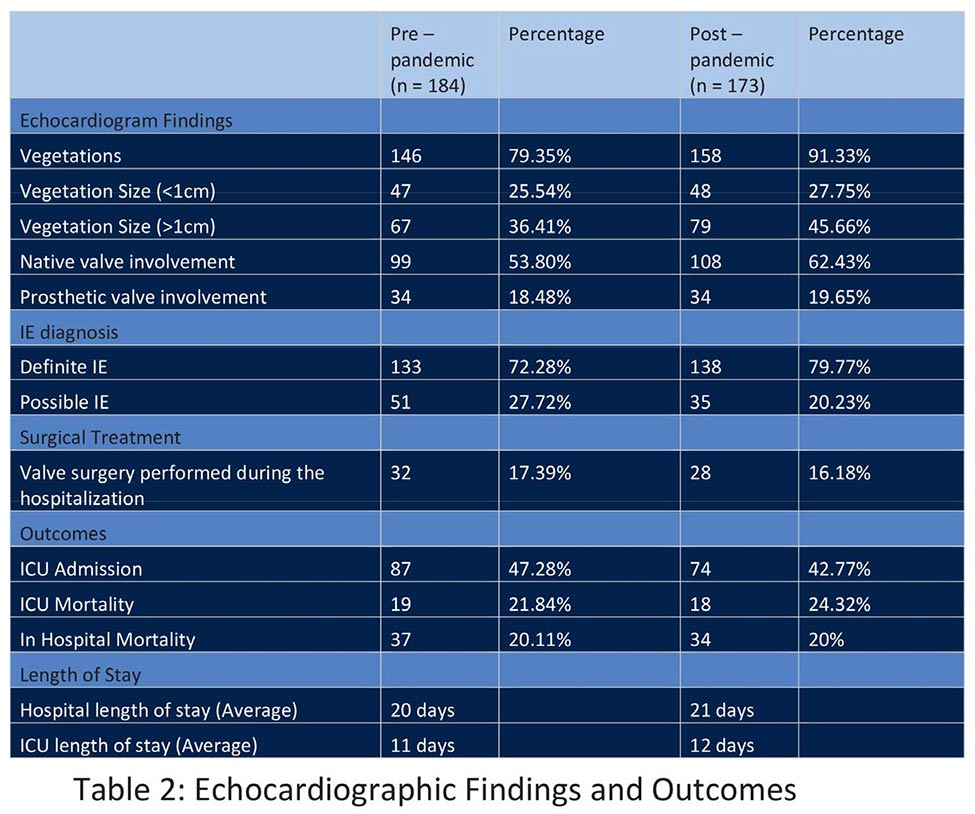

**Methods:**

We conducted a retrospective descriptive study of hospitalized adults admitted to Maimonides Health from January 1, 2016, to June 30, 2024, who are diagnosed with IE based on the 2023 Duke-ISCVID IE criteria and received at least 72 hours of antibiotics. The study population was categorized into two groups: pre-COVID pandemic (2016-2019) and post-COVID pandemic (2021-2024).

**Figure:**
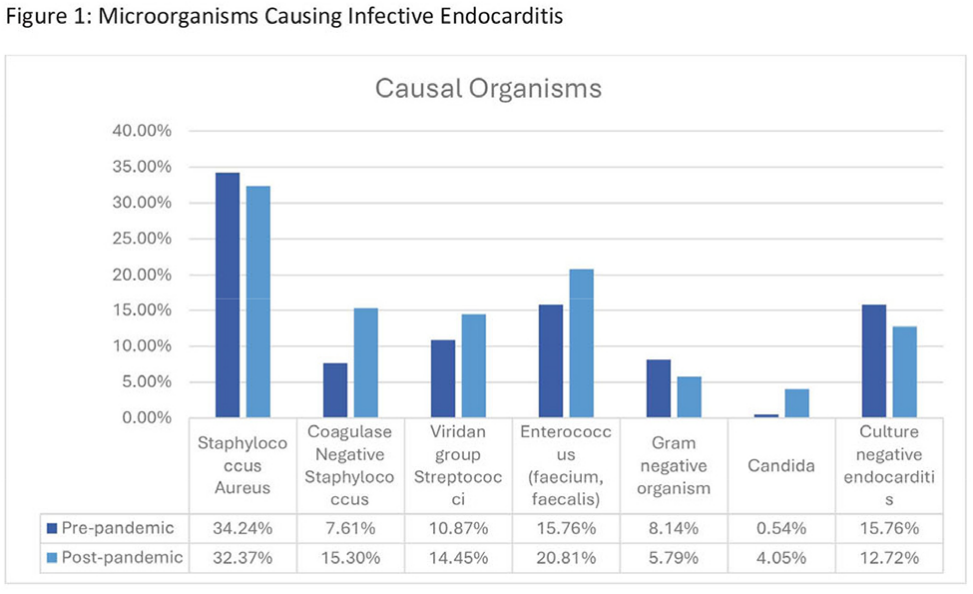

**Figure:**
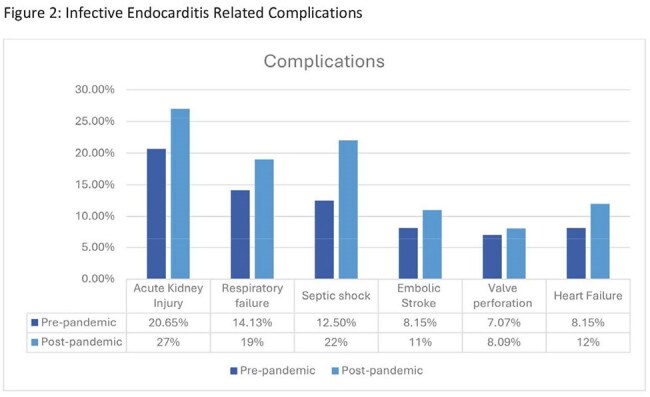

**Results:**

A total of 357 patients were included in the study, with 184 in the pre-pandemic group and 173 in the post-pandemic group. The majority were Caucasian males over the age of 50. In both groups, 50% or fewer patients presented with fever, and over 80% met SIRS criteria. In the post-pandemic group, cases trended toward later presentation, increased vegetation findings on echocardiograms, more patients with definite IE, and IE-related complications. However, incidence, in-hospital mortality, and LOS remained similar between the two groups.

**Conclusion:**

Despite innovations in diagnostics, trends in our data revealed that patients presented later, with larger vegetations, more definite IE diagnoses, and higher IE-related complications during the post-pandemic period but the incidence, in-hospital mortality, and LOS among patients with IE were comparable between the pre- and post-pandemic groups. It is possible that patients experienced a more subacute presentation, which may have delayed their seeking care. These findings highlight the complexity of IE management and underscore the potential need for earlier recognition, particularly in the outpatient setting, to facilitate timely treatment and improve patient outcomes.

**Disclosures:**

All Authors: No reported disclosures

